# Impact of high disease activity on damage accrual and disease outcomes in childhood-onset systemic lupus erythematosus

**DOI:** 10.3389/fped.2026.1765082

**Published:** 2026-06-18

**Authors:** Ninlapat Jidmahawong, Butsabong Lerkvaleekul, Kwanchai Pirojsakul, Soamarat Vilaiyuk

**Affiliations:** 1 Division of Rheumatology, Department of Pediatrics, Faculty of Medicine Ramathibodi Hospital, Mahidol University, Bangkok, Thailand; 2 Division of Nephrology, Department of Pediatrics, Faculty of Medicine Ramathibodi Hospital, Mahidol University, Bangkok, Thailand

**Keywords:** children, connective tissue disease, corticosteroids, damage, high disease activity, lupus, outcomes

## Abstract

**Objective:**

The impact of the duration and cumulative high disease activity on damage accrual in childhood-onset SLE (cSLE) is less well established. This study aimed to assess the cumulative burden of high disease activity and its association with damage accrual and clinical outcomes in patients with cSLE.

**Methods:**

This retrospective cohort study included cSLE patients with at least one year of follow-up. High disease activity status (HDAS) was defined as a SLEDAI-2 K score ≥10. Patients were classified into two groups: HDAS (ever experienced HDAS) and non-HDAS. Time-adjusted cumulative HDAS (cHDAS) was defined as the proportion of time spent in HDAS during the observation period. Clinical features, laboratory parameters, treatment, organ damage assessed by the SLICC/ACR Damage Index, and remission status were recorded at baseline and each follow-up visit. Logistic regression, Cox proportional hazards models, and Kaplan–Meier analyses were performed.

**Results:**

Among 196 cSLE (mean age at diagnosis 11.3 ± 2.9 years; 82.1% female), 126 (64.3%) experienced high disease activity status (HDAS). Patients with HDAS had a significantly higher rate of damage accrual than those without (SDI ≥ 1: 33.3% vs. 11.4%, *p* < 0.001), with an odds ratio of 3.0 (95% CI 1.2–7.3; *p* = 0.017). Time-adjusted cHDAS was significantly higher in patients with damage than in those without (11.8 [2.4–33.0] vs. 1.5 [0–5.4], *p* < 0.001). Increasing thresholds of time-adjusted cHDAS were associated with a progressively higher risk of damage accrual, with hazard ratios rising from 6.2 at ≥5% to 16.1 at ≥15% (all *p* < 0.001). The mean time to damage was significantly shorter in patients with time-adjusted cHDAS ≥5% compared with <5% (5.0 [3.7–6.2] vs. 12.5 [11.3–13.7] years, *p* < 0.001). In multivariable analysis, neuropsychiatric involvement and time-adjusted cHDAS ≥5% remained independent predictors of damage accrual, with hazard ratios of 2.4 (95% CI 1.3–4.4, *p* = 0.008) and 4.2 (95% CI 2.1–8.3, *p* < 0.001), respectively.

**Conclusions:**

Higher time-adjusted cHDAS was associated with an increased risk of damage accrual, indicating that greater cumulative exposure to high disease activity is associated with damage. Neuropsychiatric involvement and time-adjusted cHDAS ≥5% were significantly associated with damage accrual.

## 1 Introduction

Systemic lupus erythematosus (SLE) is a systemic autoimmune disease affecting multiple organ systems, and it has diverse clinical manifestations. SLE can lead to significant morbidity and mortality. Clinical manifestations and disease severity vary among patients based on the affected organ systems. Childhood-onset SLE (cSLE) is defined as disease onset before the age of 18 years. cSLE affects approximately 15%–20% of the overall SLE population, with a prevalence rate of 3.3–8.8 per 100,000 children (1). The average age at diagnosis is 11–12 years, and onset before the age of 5 years is relatively rare. The etiological, pathogenic, and laboratory features of cSLE are similar to those of adult-onset SLE. However, the two conditions differ in terms of the frequency and severity of clinical manifestations. Notably, cSLE is more likely to present with a more severe disease course, a higher frequency of renal and neuropsychiatric involvement, and poorer outcomes compared with adult SLE (2).

In SLE, assessing disease activity is essential for detecting flare-ups, monitoring therapeutic efficacy, and predicting clinical outcomes. There are several available tools for evaluating disease severity. The Systemic Lupus Erythematosus Disease Activity Index 2000 (SLEDAI-2K) is one of the most widely used indices in clinical research. Previous studies have shown that patients with a high disease activity status (HDAS) are at increased risk for severe organ involvement, particularly neuropsychiatric manifestations, renal involvement, and vasculitis. These patients are more likely to have low baseline complement levels and test positive for anti-double-stranded DNA (anti-dsDNA) and anti-Smith antibodies. They also require intensive immunosuppressive medications and are at increased risk of damage accrual and disease flare-ups (3). In addition, recurrent or prolonged periods of HDAS have been associated with younger age at disease onset, more severe clinical presentations, a greater serological activity, and a higher risk of damage accrual. A total cumulative duration of HDAS (cHDAS) of >2 years can significantly affect long-term organ damage (4). In another study, patients with an extremely high initial disease activity are less likely to achieve a low disease activity state during the disease course (5).

Despite these findings, the existing literature is largely derived from adult SLE cohorts, while studies focusing on cSLE remain limited. Furthermore, most prior research has emphasized low disease activity states and their association with improved outcomes, whereas relatively less attention has been given to the impact of high disease activity on the risk of damage. In particular, the effects of the duration and cumulative burden of high disease activity on damage accrual in cSLE have not been well established. Therefore, this study aimed to evaluate whether high disease activity, including its cumulative duration, is associated with adverse disease outcomes, particularly damage accrual, in patients with cSLE.

## 2 Materials and methods

This retrospective single-center cohort study included all patients who had been diagnosed with SLE according to the 1997 American College of Rheumatology (ACR) criteria (6), the 2012 Systemic Lupus International Collaborating Clinics criteria (7), or the 2019 European League Against Rheumatism/ACR Classification Criteria for Systemic Lupus Erythematosus (8) before the age of 18 years, and who had at least 1 year of follow-up at Ramathibodi Hospital, a tertiary referral center, between January 2008 and March 2023. Observation time was defined as the duration from cohort entry (date of diagnosis or first eligible visit at the study center) to the occurrence of disease damage or the last follow-up visit, whichever occurred first; this corresponded to time to damage for patients who developed damage and total follow-up time for those who were censored. Patients were managed through a multidisciplinary care approach involving pediatric rheumatologists and nephrologists, with routine standardized assessment of disease activity and management. Patients with incomplete medical records or a diagnosis of overlap syndrome were excluded from the study. The current study was approved by the Ethics Committee of the Faculty of Medicine, Ramathibodi Hospital, Mahidol University (MURA2024/214), and the need for informed consent was waived due to the retrospective nature of the study.

### 2.1 Data collection

#### 2.1.1 Baseline characteristics

The data of eligible patients were extracted from the medical records. Details of the demographic and baseline characteristics of the participants, including age, age at diagnosis, sex, disease duration, and duration prior to treatment initiation, were collected. The clinical manifestations included initial presenting symptoms, SLEDAI-2K score at diagnosis, and relevant laboratory findings such as complete blood count, erythrocyte sedimentation rate, complement levels, antinuclear antibody, anti-dsDNA, direct Coombs test, and antiphospholipid antibodies. Lupus nephritis (LN) classification was based on renal biopsy findings. Treatment information, including the use of medications such as hydroxychloroquine, azathioprine, methotrexate, mycophenolate mofetil, and rituximab, was recorded. Cumulative doses of prednisolone and cyclophosphamide were calculated as the sum of all administered doses over the observation period. In addition, the time-adjusted cumulative prednisolone dose was calculated by dividing the total prednisolone-equivalent dose by the total observation time. Corticosteroid doses were converted to prednisolone-equivalent doses prior to calculation. Infectious events, particularly disseminated infections, those involving major organs, or those requiring hospitalization, were documented. Data on disease flare-ups and remissions were also collected during the follow-up period.

#### 2.1.2 Disease activity assessment

Disease activity was assessed using the SLEDAI-2K, which included 24 important clinical and laboratory manifestations, with a total score of 0–105 (9). Time-adjusted mean SLEDAI-2K was used to assess disease activity over time, reflecting the average disease activity throughout follow-up. It was calculated as the area under the curve (AUC) of SLEDAI-2K over time, divided by the total duration of follow-up. The AUC was estimated using the trapezoidal rule (supplementary material) (10). The Physician Global Assessment (PGA), with a clinician-rated scale of 0–3 (11). The SLEDAI-2K and PGA scores were recorded at baseline, every 3–6 months, and at each visit during active disease or flares until the most recent follow-up visit. HDAS was defined as a SLEDAI-2K score ≥10 (3, 4). Patients were classified into two groups based on whether they ever experienced HDAS during follow-up (i.e., ≥1 visit with SLEDAI-2K ≥10), as the HDAS group and the non-HDAS group.

Cumulative HDAS (cHDAS) was defined as the total duration of time during which patients were in HDAS over the observation period. The duration of HDAS was estimated by summing time intervals between consecutive visits with SLEDAI-2K ≥10. For intervals in which SLEDAI-2K crossed the HDAS threshold between two visits, the transition time was estimated using linear interpolation, assuming a linear change between visits. To account for differences in follow-up duration across patients, time-adjusted cHDAS was calculated as the proportion of cHDAS divided by the total observation time. Time-adjusted cHDAS was used as the primary exposure variable in all analyses. The calculation formula is shown in the supplementary material.

### 2.2 Outcome assessment

#### 2.2.1 Organ damage

Organ damage was evaluated using the 2012 Systemic Lupus International Collaborating Clinics/ACR Damage Index (SDI) developed in 1996. The SDI included 12 organ systems and assigned scores based on irreversible damage that had been present for at least 6 months, whether due to disease activity, treatment, or complications, with a maximum total score of 47 (12). The patients in this study received routine eye exams every 6–12 months.

#### 2.2.2 Disease flare-up

Flare-ups were classified as mild-to-moderate or severe. A mild-to-moderate flare-up was defined by an increase in the SLEDAI-2K score to ≥3; new or worsening involvement of the skin, mucous membranes, serositis, arthritis, or fever; an increase in PGA score to 1–2.5; a slight increase in prednisolone dose (< 0.5 mg/kg/day), or addition of nonsteroidal anti-inflammatory drugs or hydroxychloroquine. A severe flare-up was defined by an increase in the SLEDAI-2K score to >12; new or worsening neuropsychiatric symptoms, vasculitis, nephritis, myositis, thrombocytopenia (platelet count <60,000/μL), and hemolytic anemia with hemoglobin levels <7 g/dL; a significant increase in prednisolone dosage (>2 times at baseline or >0.5 mg/kg/day); hospitalization due to disease activity; addition of a new immunosuppressive agent; or a PGA score of >2.5 (13).

#### 2.2.3 Low disease activity

Low disease activity was examined using three definitions: minimal disease activity (MDA), low disease activity (LDA), and lupus low disease activity state (LLDAS). MDA was defined as a clinical SLEDAI-2K score ≤1 (excluding serologic activity such as anti-dsDNA positivity and/or low complement levels), maintained with antimalarials, standard immunosuppressive doses (not exceeding the recommended doses), and low-dose prednisolone (≤5 mg/day). Only hematologic abnormalities directly attributed to lupus, such as isolated leukopenia and thrombocytopenia, were allowed under this definition (14). LDA was defined as SLEDAI-2K scores ≤2, with or without serologic activity, and limited to the presence of only one of the following conditions: rash, alopecia, mucosal ulcers, pleurisy, pericarditis, fever, thrombocytopenia, or leukopenia. Patients meeting the LDA criteria were treated with antimalarials only, without glucocorticoids or immunosuppressants (15). LLDAS was defined as a SLEDAI-2K score ≤4, with no active renal, central nervous system, cardiopulmonary, gastrointestinal, or vascular involvement; no fever or hemolytic anemia; and no new lupus disease activity compared with the previous visit. It also required a PGA score of ≤1, current treatment with prednisolone (or equivalent) at a dose of ≤7.5 mg/day, and well-tolerated standard maintenance doses of immunosuppressive and/or approved biologic agents, excluding investigational drugs (16).

#### 2.2.4 Disease remission

Disease remission was categorized into remission on therapy, remission off therapy, and complete remission. Remission on therapy was defined as a clinical SLEDAI-2K score of 0, a PGA score of <0.5, a prednisolone dose of ≤5 mg/day or ≤0.15 mg/kg/day, and the absence of escalation or the addition of immunosuppressive or biologic therapy. Remission off therapy was defined as clinical remission without systemic treatments for SLE, other than maintenance antimalarial therapy. Complete remission was defined as the achievement of clinical and serological remission (normal anti-dsDNA and complement levels) without the use of immunosuppressive agents or glucocorticoids, other than ongoing antimalarial treatment (17, 18).

### 2.3 Statistical analysis

Descriptive statistics were reported as mean (standard deviation, SD) or median (interquartile range, IQR). The Mann–Whitney *U*-test and the chi-square test were used to compare between-group differences. Flare rates were analyzed using Poisson regression models with a log link function, with the number of flares as the dependent variable and follow-up time included as an offset term to account for varying observation periods. Incidence rate ratios (IRRs) with 95% confidence intervals were reported to compare flare rates between groups. Logistic regression analysis was performed to identify factors associated with ever experiencing HDAS. Candidate variables were selected based on clinical relevance and evidence from prior literature, including demographic characteristics (age, sex, disease duration), clinical factors (damage accrual), and treatment-related variables (e.g., cumulative glucocorticoid exposure and history of infection). Variables that are components of the SLEDAI-2K score were not included in the model to avoid circularity. Kaplan–Meier survival analysis and Cox proportional hazards models were conducted to evaluate factors affecting the occurrence of damage accrual. Variables included in the Cox proportional hazards model were selected based on clinical relevance and factors associated with damage accrual in univariable analyses. The number of covariates included in the model was limited according to the number of outcome events to avoid overfitting. In addition, variables with high collinearity in logistic regression and the Cox proportional hazards model were excluded based on correlation analysis or clinical judgment. All statistical analyses were performed using the Statistical Package for the Social Sciences software version 29.0.1.0. (IBM Corp., Armonk, NY, the USA). A *p*-value of <0.05 was considered statistically significant.

## 3 Results

In total, 230 patients were diagnosed with cSLE, and 34 patients with incomplete medical histories were excluded from the analysis. Of the 196 patients with SLE in this study, 161 (82.1%) were female. The mean age at diagnosis was 11.3 ± 2.9 years, the median disease duration was 3.4 (IQR 1.8–6.1) years, and the median duration before diagnosis was 1.2 (IQR 0.5–3.2) months. The most common manifestations were hematologic involvement (71.4%) and mucocutaneous organ involvement (62.2%). The other clinical features commonly found in this cohort included renal condition, fever, vasculitis, serositis, and neurological involvement. The median baseline SLEDAI-2K and time-adjusted mean SLEDAI-2K score were 10.5 (IQR 6.0–17.0) and 2.0 (IQR 0.8–3.4).

A total of 126 (64.3%) patients had HDAS. The median time-adjusted cHDAS was 6.1 (IQR 2.8–12.5). The HDAS and non-HDAS groups did not differ significantly in sex, age, or disease duration. Almost all patients with neurological involvement had HDAS. In addition, the HDAS group had a significantly higher frequency of renal involvement, particularly LN class III-V, serositis, vasculitis, mucocutaneous and musculoskeletal involvement, compared with the non-HDAS group. In terms of laboratory results, the HDAS group had significantly lower white blood cell counts, absolute lymphocyte counts, complement levels, and a higher erythrocyte sedimentation rate, and was more likely to present with anti-dsDNA antibody positivity than the non-HDAS group. Almost all patients in both groups were treated with prednisolone (99.5%) and hydroxychloroquine (95.9%), with no significant differences between the two groups. In addition, no chloroquine was used in this study. However, the HDAS group had a higher frequency of use of azathioprine, mycophenolate mofetil, and cyclophosphamide. Moreover, they received a higher median cumulative dose of prednisolone and cyclophosphamide than the non-HDAS group (Table 1). We further evaluated factors associated with HDAS and found that a time-adjusted cumulative prednisolone dose >0.15 mg/kg/day and the presence of damage accrual were independently associated with HDAS (Table 2).

**Table 1:** Baseline characteristics of cSLE patients with and without high disease activity.

Characteristics	All patients	HDAS	Non-HDAS	*p*-value
	(*n* = 196)	(*n* = 126)	(*n* = 70)	
Female, *n* (%)	161 (82.1)	103 (81.7)	58 (82.9)	0.846
Age at diagnosis^a^	11.3 ± 2.9	11.4 ± 2.6	11.1 ± 3.3	0.480
Duration of disease (years)	3.4 (1.8–6.1)	3.0 (1.5–5.6)	4.2 (2.4–6.6)	0.093
Duration before diagnosis (months)	1.2 (0.5–3.2)	1.0 (0.5–2.3)	1.4 (0.6–3.6)	0.036*
Number of visits	17 (12–26)	16 (11–24)	18 (13–31)	0.143
Clinical manifestation, *n* (%)				
- Fever	51 (26.0)	35 (27.8)	16 (22.9)	0.50
- Hematological	140 (71.4)	91 (70.0)	49 (72.2)	0.744
- Mucocutaneous	122 (62.2)	85 (67.5)	37 (52.9)	0.047*
- Neurological	31 (15.4)	29 (23.0)	1 (1.4)	<0.001*
- Serositis	33 (16.8)	31 (24.6)	2 (2.9)	<0.001*
- Vasculitis	31 (15.8)	28 (22.2)	3 (4.3)	<0.001*
- Lupus nephritis^b^	106 (54.1)	93 (73.8)	13 (18.6)	<0.001*
- Lupus nephritis class III-V	71 (36.2)	65 (51.6)	6 (8.6)	<0.001*
- Musculoskeletal	69 (35.2)	49 (38.9)	15 (21.4)	0.017*
- Gastrointestinal	11 (5.6)	8 (6.3)	3 (4.3)	0.749
SLEDAI-2K at diagnosis	10.5 (6.0–17.0)	15.0 (12.0–19.0)	6.0 (4.0–8.0)	<0.001*
Time-adjusted mean SLEDAI-2K	2.0 (0.8–3.4)	2.4 (1.3–4.0)	1.0 (0.2–2.0)	<0.001*
Laboratory				
Hematocrit^a^	30.6 ± 7.0	30.7 ± 5.8	30.2 ± 8.9	0.866
WBC (×10^3^ cells/mm^3^)	5.4 (3.7–8.3)	5.0 (3.3–7.2)	6.0 (4.7–8.9)	<0.002*
Absolute lymphocyte count (×10^3^ cells/mm^3^)	1.6 (1.1–2.4)	1.3 (0.9–2.0)	1.9 (1.3–2.8)	<0.001*
Leukopenia/lymphopenia, *n* (%)	84 (45.7)	64 (54.7)	20 (29.9)	0.001*
Platelet (×10^3^ cells/mm^3^)	219 (137–311)	214 (132–297)	231 (99–336)	0.477
Thrombocytopenia, *n* (%)	45 (24.1)	26 (21.7)	19 (28.4)	0.305
Erythrocyte sedimentation rate (mm/h)	60 (28–87)	65 (35.5–88)	53.5 (22–77.5)	0.051
C3 (g/L)	0.57 (0.31–0.95)	0.40 (0.27–0.74)	0.95 (0.65–1.24)	<0.001*
C4 (g/L)	0.07 (0.03–0.16)	0.06 (0.03–0.10)	0.16 (0.07–0.27)	<0.001*
Hypocomplementemia, *n* (%)	136 (73.9)	105 (89.0)	31 (47.0)	<0.001*
Anti-dsDNA, *n* (%)	134 (74.0)	104 (90.4)	30 (45.5)	<0.001*
Direct Coombs' test, *n* (%)	94 (62.3)	63 (64.9)	31 (57.4)	0.360
Antiphospholipid antibodies, *n* (%)	26 (16.7)	18 (18.9)	8 (13.1)	0.340
Treatment				
Hydroxychloroquine, *n* (%)	188 (95.9)	120 (95.2)	68 (97.1)	0.714
Prednisolone, *n* (%)	195 (99.5)	126 (100.0)	69 (98.6)	0.357
Cumulative dose of prednisolone (mg/kg)	265.6 (171–459)	317.2 (199.9–487.0)	220.2 (136.1–343.6)	<0.001*
Time-adjusted cumulative prednisolone dose	0.26 (0.15–0.48)	0.29 (0.21–0.53)	0.17 (0.07–0.29)	<0.001*
Cyclophosphamide, *n* (%)	91 (46.4)	83 (65.9)	8 (11.4)	<0.001*
Cumulative dose of cyclophosphamide (mg/m^2^)	0 (0–5,665)	4,313 (0–6,140.3)	0 (0–0)	<0.001*
Mycophenolate mofetil, *n* (%)	46 (23.5)	33 (26.2)	13 (18.6)	0.228
Azathioprine, *n* (%)	139 (70.9)	96 (76.2)	43 (61.4)	0.029*
Methotrexate, *n* (%)	12 (6.1)	8 (6.3)	4 (5.7)	1.00
Cyclosporine, *n* (%)	5 (2.6)	2 (1.6)	3 (4.3)	0.351
Immunoglobulin, *n* (%)	9 (4.6)	7 (5.6)	2 (2.9)	0.495
Rituximab, *n* (%)	4 (2.0)	2 (1.6)	2 (2.9)	0.618

Data was presented as median (P25–75);.

a mean ± SD.

* *p* < 0.05 was set as significance.

HDAS, High disease activity status; SLEDAI-2K, The Systemic Lupus Erythematosus Disease Activity Index 2000; WBC, white blood cell; C, complement component; Anti-dsDNA, anti-double stranded DNA.

b Among patients with lupus nephritis, renal biopsy was performed in 74/93 (79.6%) in the HDAS group and 9/13 (69.2%) in the non-HDAS group.

**Table 2:** Factors associated with ever experiencing high disease activity status: logistic regression analysis.

Factors	Univariable analysis		Multivariable analysis	
	OR (95%CI)	*p-*value	OR (95%CI)	*p-*value
Gender	0.9 (0.4–2.0)	0.846	0.9 (0.4–2.0)	0.718
Age at diagnosis (year)	1.0 (0.9–1.1)	0.479	1.1 (1.0–1.2)	0.121
Disease duration	0.9 (0.9–1.0)	0.185	1.1 (1.0–1.2)	0.181
Time-adjusted cumulative prednisolone dose >0.15 mg/kg/day	3.8 (1.9–7.4)	<0.001*	4.3 (1.9–9.7)	<0.001*
Damage accrual	1.9 (1.3–2.7)	<0.001*	3.0 (1.2–7.3)	0.017*
Infection	2.9 (1.4–5.9)	0.004*	2.0 (0.9–4.4)	0.072

* *p* < 0.05.

### 3.1 Disease outcomes

**Figure 1:**
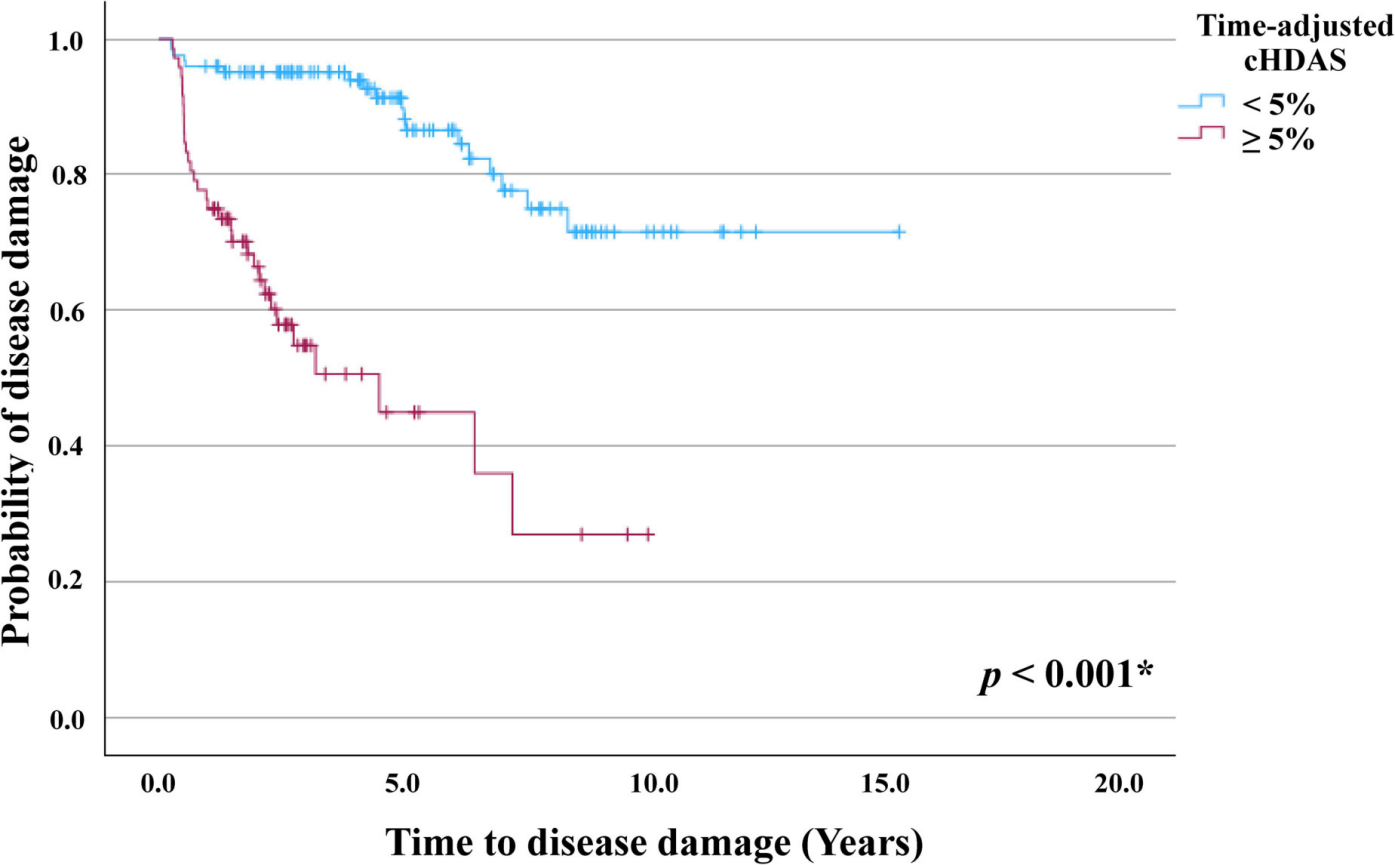
Kaplan–meier analysis of time to disease damage in cSLE patients stratified by time-adjusted cumulative high disease activity status (cHDAS) (≥5% vs. <5%).

Among the 196 patients with cSLE, 50 (25.5%) developed organ damage. The most frequently affected systems were ocular and neuropsychiatric, followed by musculoskeletal damage. Cataract accounted for 81.3% of all ocular complications, and the frequency of glaucoma (12.5%), retinal scar (6.3%), and central scotoma (6.3%) was lower. Neuropsychiatric involvement comprised seizures requiring prolonged therapy (43.8%), cerebrovascular accidents (37.5%), and cognitive impairment or major psychosis (37.5%) (Supplementary Table S1). In our cohort, all patients had no end-organ damage at baseline. Damage accrual was more common in the HDAS group than in the non-HDAS group (42 [33.3%] vs. 8 [11.4%], *p* = <0.001). Ocular damage occurred significantly more often in the HDAS group than in the non-HDAS group (14 [11.1%] vs. 2 [2.9%], *p* = 0.043) (Table 3). Then, we assessed whether high disease activity was associated with damage accrual by using time-adjusted cHDAS. The median time-adjusted cHDAS differed significantly between the SDI and non-SDI groups (11.8 (IQR 2.4–33.0) vs. 1.5 (IQR 0–5.4), *p* < 0.001). Moreover, higher time-adjusted cHDAS thresholds were consistently associated with an increased risk of damage accrual (SDI ≥ 1). Specifically, patients with time-adjusted cHDAS ≥ 5% had a 6.2-fold higher risk of damage accrual, which increased progressively to 8.4-fold at ≥7.5%, 12.2-fold at ≥10%, and 16.1-fold at ≥15%. All associations were statistically significant (*p* < 0.001; see Table 4). Furthermore, the mean time to damage accrual was significantly shorter in patients with time-adjusted cHDAS ≥5% than in those with time-adjusted cHDAS <5% (5.0 years (3.7–6.2) vs. 12.5 years (11.3–13.7), *p* < 0.001) (Figure 1). Similarly, higher percentages of time-adjusted cHDAS were associated with a shorter time to damage accrual (Table 5).

**Table 3:** Disease outcomes of cSLE patients with and without high disease activity.

Disease damage	All patients	HDAS	Non-HDAS	*p*-value
	(*n* = 196)	(*n* = 126)	(*n* = 70)	
SDI, *n* (%)	50 (25.5)	42 (33.3)	8 (11.4)	<0.001*
- Ocular	16 (8.2)	14 (11.1)	2 (2.9)	0.043*
- Neuropsychiatric	16 (8.2)	13 (10.3)	3 (4.3)	0.178
- Renal	2 (1.0)	2 (1.6)	0	0.538
- Pulmonary	3 (1.5)	3 (2.4)	0 (0)	0.554
- Cardiovascular	0	0	0	-
- Peripheral vascular	1 (0.5)	1 (0.8)	0	1
- Musculoskeletal	14 (7.1)	12 (9.5)	2 (2.9)	0.082
- Skin	2 (1.0)	0	2 (2.9)	0.126
- Gastrointestinal	0	0	0	-
- Gonadal failure	1 (0.5)	1 (0.8)	0	1
- Diabetes mellitus	5 (2.6)	5 (4.0)	0	0.162
- Malignancy	1 (0.5)	1 (0.8)	0	1
Infection, *n* (%)	59 (30.1)	47 (37.3)	12 (17.1)	0.003*
Flare, *n* (%)	78 (39.8)	48 (38.1)	30 (42.9)	0.514
Flare rate (per 100 person-years)	17.1	16.8	17.6	–
Incidence rate ratio for total flares^a^	0.95 (95% CI 0.68–1.33)			0.772
Mild to moderate flare rate (per 100 person-year)	11.3	9.2	14.5	-
Incidence rate ratio for mild to moderate flares^a^	0.63 (95% CI 0.42–0.95)			0.027*
Severe flare rate (per 100 person-year)	5.8	7.6	3.1	-
Incidence rate ratio for severe flares^a^	2.45 (95% CI 1.22–4.93)			0.012*
Duration before flare (years)	3.0 (1.4–5.1)	3.0 (1.5–5.0)	2.8 (0.9–5.6)	0.606
Low disease activity and remission, *n* (%)				
LLDAS	141 (71.9)	84 (66.7)	57 (81.4)	0.028*
LDA	43 (21.9)	16 (12.7)	27 (38.6)	<0.001*
MDA	142 (72.4)	84 (66.7)	58 (82.9)	0.015*
Remission on medication	123 (62.8)	75 (59.5)	48 (68.6)	0.209
Remission off medication	38 (19.4)	14 (11.1)	24 (34.3)	<0.001*
Complete remission	23 (11.7)	7 (5.6)	16 (22.9)	<0.001*

* *p* < 0.05 was set as significance,.

SDI, Systemic Lupus International Collaborating Clinics/American College of Rheumatology (SLICC/ACR) Damage Index; MDA, minimal disease activity; LDA, low disease activity; LLDAS, lupus low disease activity.

a Incidence rata ratio for total flares, mild to moderate flares, and severe flares using the non-HDAS group as a reference.

**Table 4:** The association between time-adjusted cumulative high disease activity (cHDAS) and damage accrual (SDI ≥ 1).

Time-adjusted cHDAS	Hazard Ratio (95%CI)	*p*-value
≥5%	6.2 (3.4–11.3)	<0.001*
≥7.5%	8.4 (4.6–15.3)	<0.001*
≥10%	12.2 (6.6–22.6)	<0.001*
≥15%	16.1 (8.4–30.9)	<0.001*

* A *p* < 0.05 was considered statistically significant.

SDI, Systemic Lupus International Collaborating Clinics/American College of Rheumatology Damage Index; CI, confidence interval.

**Table 5:** The kaplan–meier analysis related to the percentage of time-adjusted cHDAS and the development of damage accrual (SDI ≥ 1).

% Time-adjusted cHDAS	Mean time to disease damage (years)	Median time to disease damage (years)	*p*-value
≥5%	5.0 (95% CI 3.7–6.2)	4.6 (95% CI 1.5–7.7)	<0.001*
≥7.5%	3.9 (95% CI 2.6–5.1)	2.2 (95% CI 1.6–2.8)	<0.001*
≥10%	2.8 (95% CI 1.8–3.8)	1.5 (95% CI 0.4–2.6)	<0.001*
≥15%	1.5 (95% CI 0.8–2.2)	0.6 (95% CI 0.3–0.8)	<0.001*

* *p* < 0.05 was set as significance; cHDAS, cumulative high disease activity status; Time-adjusted cHDAS, cHDAS divided by disease duration; SDI, Systemic Lupus International Collaborating Clinics/American College of Rheumatology Damage Index.

Among the patients in this cohort, 141 (71.9%) achieved low disease activity as defined by LLDAS. Additionally, 123 (62.8%) achieved remission on medication, while 38 (19.4%) achieved remission off medication; 23 patients (11.7%) met criteria for complete remission. Infections occurred in 59 patients (30.1%), with a higher proportion observed in the HDAS group compared with the non-HDAS group (37.3% vs. 17.1%, *p* = 0.003). Overall, 78 patients (39.8%) experienced disease flares. The overall flare rate was similar between groups (16.8 vs. 17.6 per 100 person-years; IRR 0.95, 95% CI 0.68–1.33, *p* = 0.772). However, when stratified by severity, the rate of mild-to-moderate flares was lower in the HDAS group (9.2 vs. 14.5 per 100 person-years; IRR 0.63, 95% CI 0.42–0.95, *p* = 0.027), whereas the rate of severe flares was higher (7.6 vs. 3.1 per 100 person-years; IRR 2.45, 95% CI 1.22–4.93, *p* = 0.012), as shown in Table 3.

### 3.2 Predictors of damage accrual in patients with childhood-onset SLE

In the multivariable analysis, after adjustment for confounders, neuropsychiatric SLE (NPSLE) and time-adjusted cHDAS ≥5% remained independent predictors of damage accrual, with hazard ratios of 2.4 (95% CI 1.3–4.4, *p* = 0.008) and 4.2 (95% CI 2.1–8.3, *p* < 0.001), respectively. In contrast, time-adjusted cumulative prednisolone dose ≤0.15 mg/kg/day was no longer significantly associated with damage accrual (HR 0.5, 95% CI 0.2–1.3, *p* = 0.167) (Table 6). To further delineate the independent effects of glucocorticoid exposure from disease activity, additional analyses were performed to evaluate the association between time-adjusted cumulative prednisolone dose and damage accrual separately from HDAS. In these analyses, a higher time-adjusted cumulative prednisolone dose was associated with a shorter time to damage accrual in Kaplan–Meier analysis, and with an increased risk of damage accrual in univariable analyses (Supplementary Tables S2, S3).

**Table 6:** Factors associated with damage accrual: Cox proportional hazards analysis.

Factors	Univariable analysis		Multivariable analysis	
	HR (95%CI)	*p*-value	HR (95%CI)	*p*-value
Age at diagnosis	1.0 (0.9–1.1)	0.924	1.0 (0.9–1.1)	0.751
Female	1.1 (0.5–2.3)	0.836	1.4 (0.6–3.0)	0.418
NPSLE	3.2 (1.8–5.9)	<0.001*	2.4 (1.3–4.4)	0.008*
Time-adjusted cHDAS ≥5%	6.2 (3.4–11.3)	<0.001*	4.2 (2.1–8.3)	<0.001*
Time-adjusted cumulative prednisolone dose ≤0.15 mg/kg/day	0.3 (0.1–0.6)	0.002*	0.5 (0.2–1.3)	0.167

* *p* < 0.05 was set as significance.

cHDAS, cumulative high disease activity status; Time-adjusted cHDAS, cHDAS divided by disease duration. HR, hazard ratio; CI, confidence interval.

## 4 Discussion

Understanding the impact of disease activity on disease outcomes is essential in the management of cSLE. Persistent high disease activity and treatment-related adverse effects may contribute to irreversible organ damage; therefore, evaluating the role of HDAS, particularly its cumulative burden, is critical for optimizing long-term care. In this study, patients with HDAS had more severe disease phenotypes, greater cumulative glucocorticoid exposure, and were less likely to achieve low disease activity or remission, resulting in poorer outcomes, including infections and severe flares. Furthermore, increasing levels of time-adjusted cHDAS were associated with a progressively higher risk of damage accrual and a shorter time to damage. In multivariable analysis, time-adjusted cHDAS ≥ 5% and NPSLE were identified as independent predictors of damage accrual. These findings suggest that cumulative burden of high disease activity plays a critical role in disease outcomes in cSLE.

In this study, we defined HDAS as a SLEDAI-2K score ≥10, based on previous literature demonstrating that this threshold identifies patients with more severe disease, greater treatment burden, and a higher risk of damage accrual (3, 4, 19, 20). To assess the impact of high disease activity on outcomes, patients were classified according to whether they had ever experienced HDAS during the observation period, rather than relying only on baseline disease activity, thereby better reflecting the longitudinal disease course. Given that cumulative disease activity over time is a strong predictor of damage accrual (21), we extended this concept to cumulative high disease activity. To quantify this burden, we used time-adjusted cHDAS, defined as the proportion of follow-up time spent in HDAS. This approach provides a more comprehensive assessment of sustained high disease activity and, importantly, normalizes for differences in follow-up duration across patients, enabling more reliable comparisons.

Our findings are consistent with those of previous studies (3, 4), which showed that patients with HDAS were more likely to present with NPSLE, LN, serositis, and that positive anti-dsDNA antibodies and low complement levels were also associated with HDAS. Although these studies were conducted primarily on adult populations, the associations were similar in our pediatric cohort. This is biologically plausible, as patients with more severe lupus were more likely to test positive for anti-dsDNA and hypocomplementemia, which reflect a high disease activity (3). Although the proportion of female patients in our cohort was slightly lower than that reported in some SLE cohorts, it remained within the expected range for cSLE, where females account for approximately 75%–90% of cases, with increasing female predominance during adolescence (22–26). The pattern of organ involvement was comparable to that reported in previous studies, with hematologic, mucocutaneous, and musculoskeletal manifestations being the most common, followed by LN (approximately 40%) and NPSLE (approximately 10%–15%) (22, 23, 26).

In this study, approximately two-thirds of patients in this cohort had HDAS. The main factors associated with HDAS were time-adjusted cumulative prednisolone dose >0.15 mg/kg/day and presence of damage accrual. During periods of HDAS, patients more frequently exhibited severe clinical features, including NPSLE, renal involvement (especially LN class III–V), serositis, and vasculitis. This higher disease burden likely required higher cumulative prednisolone use and may have contributed to subsequent damage accrual.

The most frequent damage to the system/organ in patients with HDAS in this study differed from that reported in previous ones (3). Based on an earlier study, the main associated with HDAS was renal damage accrual. Meanwhile, our findings showed that the predominant damage accrual was ocular complications, and there was no significant association with renal outcomes. This discrepancy may be explained by differences in study populations. In a previous study (3), most patients were adults, with approximately half of them being older than 45 years. In elderly patients, LN often developed >5 years after diagnosis, and it was compounded by comorbidities such as hypertension, leading to chronic renal damage, and was more challenging to manage (27). In contrast, most children with cSLE present with LN at diagnosis (28, 29) and typically receive early and aggressive treatment, which may prevent long-term renal damage. Ocular damage is likely related to corticosteroid use. This is because corticosteroids alter the biological structure of the trabecular meshwork and increase intraocular pressure (30–32). Moreover, children are particularly susceptible because their trabecular meshwork is immature (33). Thus, they are vulnerable to steroid-induced ocular complications even at lower doses and shorter treatment durations compared with adults (34).

Although our results demonstrated that HDAS was associated with damage accrual, the cumulative burden and timing of cHDAS had not been fully explored. Therefore, we further evaluated multiple time-adjusted cHDAS thresholds as predictors of damage accrual, while adjusting for potential confounders in a multivariable analysis. We found that the presence of NPSLE and time-adjusted cHDAS ≥5% were independently associated with an increased risk of damage accrual. These findings are consistent with previously identified risk factors for damage accrual (3, 4, 21, 35–37). However, most prior studies have quantified HDAS based on the number of episodes rather than its cumulative burden over time. In contrast, our approach incorporates the longitudinal burden of HDAS, which may better reflect the overall disease course in patients with cSLE.

Patients with NPSLE had a higher burden, as reflected in the SLEDAI-2K score, which is also related to HDAS. NPSLE commonly presents with psychosis and seizures, which reasonably account for complications such as cognitive impairment, seizure requiring prolonged therapy, and cerebrovascular accidents. Antiphospholipid antibodies can increase the risk of venous and arterial thrombosis (38), and a previous study showed that they play a role in thrombotic damage (39), particularly in NPSLE. However, in our cohort, only three patients with NPSLE tested positive for antiphospholipid antibodies, of whom one presented with thrombosis. The patients who tested negative for these antibodies also developed cerebrovascular accidents. This finding indicated that the role of antiphospholipid antibodies in organ damage among patients with NPSLE remains controversial, as thrombotic events in SLE are influenced by multiple factors, not only antiphospholipid antibodies. In addition to high disease activity and neurological involvement, which were associated with damage accrual in this study, a previous study had also highlighted severe disease flares as a factor associated with increased damage accrual (40). Although our patients with HDAS experienced a significantly higher incidence of severe flares compared with those without HDAS, severe flares were not significantly associated with damage accrual in univariable analysis. This suggests that, while severe flares are more frequent in patients with high disease activity, they may not independently predict damage accrual in our cohort. The difference between the previous study and this study may reflect variations in analytical approach. A previous study primarily used Chi-square or Fisher's exact tests, whereas we applied Cox proportional hazards analysis to account for time-to-event data; however, as the association was not significant in univariable analysis, it was not carried forward into the multivariable model.

Corticosteroids remain indispensable in the management of cSLE; they are also a major contributor to organ damage (21, 41, 42), including cataracts, avascular necrosis, osteoporotic fractures, and coronary artery disease (43). In this study, the time-adjusted cumulative prednisolone dose was statistically significant in univariable analysis but not in the multivariable model. This attenuation may be explained by confounding, as patients with higher disease activity are more likely to receive greater cumulative corticosteroid exposure. To further explore this relationship, we conducted an additional analysis examining the effect of increasing cumulative prednisolone dose on damage accrual. We observed a dose–response relationship, in which higher time-adjusted cumulative prednisolone doses were associated with both a shorter time to, and an increased risk of, damage accrual.

Approximately 7% of patients developed musculoskeletal damage, most commonly avascular necrosis, followed by osteoporotic fractures and joint deformities. The median time to avascular necrosis was 2.7 years, which is shorter than that reported in adult-onset SLE (aSLE) studies (44–50). A likely explanation is that our patients were younger at disease onset, a known risk factor for avascular necrosis in SLE (51–53). Based on previous studies early-onset avascular necrosis can occur in patients with SLE even with a lower cumulative corticosteroid exposure. These patients develop avascular necrosis more frequently compared with those with other rheumatic diseases (54, 55). This finding may be explained by the disease itself. This is because SLE is associated with vascular involvement and an increased risk of thrombosis due to alterations in coagulation, particularly in patients with LN (56, 57).

Previous studies have demonstrated that longer disease duration is associated with an increased risk of organ damage (35, 36, 58), and many have emphasized that achieving LLDAS is associated with reduced long-term damage (59–64). Building on this concept, we propose that the cumulative burden of high disease activity should also be considered, as failure to promptly control high disease activity may independently contribute to damage accrual. Consistent with this hypothesis, this study showed that patients with cSLE who had a time-adjusted cHDAS ≥5% had approximately a 4-fold higher risk of damage accrual compared with those with a time-adjusted cHDAS <5%.

This study has several limitations. It included a relatively small sample size, employed a retrospective design, and had a moderate follow-up duration, which may have led to an underestimation of damage accrual. In addition, it was conducted at a single tertiary care center, potentially limiting data completeness and generalizability. Therefore, prospective multicenter studies are warranted to validate these findings. Despite these limitations, the study has notable strengths. The longitudinal assessment of disease activity and treatment exposure using time-adjusted measures, including SLEDAI-2K, cHDAS, and prednisolone dose, provides a more accurate representation of real-world disease burden than cross-sectional analyses. Furthermore, the systematic collection of clinical, laboratory, and treatment data at 3–6 month intervals and at each visit during active disease or flares enabled the development of a comprehensive longitudinal dataset. These features strengthen the reliability of our evaluation of clinical outcomes, including organ damage and treatment effects.

## 5 Conclusion

Our study highlights the importance of close monitoring and optimal disease management in patients with cSLE who experience HDAS. Prolonged exposure to high disease activity was strongly associated with both an increased risk of, and a shorter time to, damage accrual. Notably, patients with NPSLE were identified as having a significantly higher risk of damage accrual. These findings underscore the critical need for early, sustained, and effective disease control to minimize long-term complications and improve overall patient outcomes.

## Data Availability

The original contributions presented in the study are included in the article/Supplementary Material, further inquiries can be directed to the corresponding author.

## References

[1] Tang SP Lim SC Arkachaisri T. Childhood-Onset systemic lupus erythematosus: southeast Asian perspectives. J Clin Med. (2021) 10:559. 10.3390/jcm10040559 33546120 PMC7913223

[2] Hoffman IE Lauwerys BR Keyser F Huizinga TW Isenberg D Cebecauer L. Juvenile-onset systemic lupus erythematosus: different clinical and serological pattern than adult-onset systemic lupus erythematosus. Ann Rheum Dis. (2009) 68(3):412–5. 10.1136/ard.2008.094813 18930995

[3] Koelmeyer R Nim HT Nikpour M Sun YB Kao A Guenther O. High disease activity status suggests more severe disease and damage accrual in systemic lupus erythematosus. Lupus Sci Med. (2020) 7:e000372. 10.1136/lupus-2019-000372 32467293 PMC7259842

[4] Hoi A Koelmeyer R Bonin J Sun Y Kao A Gunther O. Disease course following high disease activity status revealed patterns in SLE. Arthritis Res Ther. (2021) 23:191. 10.1186/s13075-021-02572-1 34261522 PMC8278658

[5] Peschken CA Wang Y Abrahamowicz M Pope J Silverman E Sayani A. Persistent disease activity remains a burden for patients with systemic lupus erythematosus. J Rheumatol. (2019) 46:166. 10.3899/jrheum.171454 30219771

[6] Hochberg MC. Updating the American college of rheumatology revised criteria for the classification of systemic lupus erythematosus. Arthritis Rheum. (1997) 40(9):1725. 10.1002/art.1780400928 9324032

[7] Petri M Orbai A-M Alarcón GS Gordon C Merrill JT Fortin PR. Derivation and validation of systemic lupus international collaborating clinics classification criteria for systemic lupus erythematosus. Arthritis Rheum. (2012) 64(8):2677–86. 10.1002/art.34473 22553077 PMC3409311

[8] Aringer M Costenbader KH Daikh DI Brinks R Mosca M Ramsey-Goldman R. 2019 EULAR/ACR classification criteria for systemic lupus erythematosus. Arthritis Rheumatol. (2019) 71(9):1400–12. 10.1002/art.40930 31385462 PMC6827566

[9] Gladman DD Ibanez D Urowitz MB. Systemic lupus erythematosus disease activity Index 2000. J Rheumatol. (2002) 29:2. 11838846

[10] Brunner HI Silverman ED Bombardier C Feldman BM. European Consensus lupus activity measurement is sensitive to change in disease activity in childhood-onset systemic lupus erythematosus. Arthritis Care Res (Hoboken). (2003) 49(3):335–41. 10.1002/art.11111 12794788

[11] Piga M Chessa E Morand EF Ugarte-Gil MF Tektonidou M Vollenhoven RV. Physician global assessment (PGA) international standardization COnsensus in systemic lupus erythematosus: the PISCOS study. Lancet Rheumatol. (2022) 4(6):e441–e9. 10.1016/S2665-9913(22)00107-2 38293958

[12] Ghazali WSW Daud SMM Mohammad N Wong KK. Slicc damage index score in systemic lupus erythematosus patients and its associated factors. Medicine (Baltimore). (2018) 97(42):e12787. 10.1097/MD.0000000000012787 30334968 PMC6211909

[13] Ruperto N Hanrahan L Alarcón G Belmont H Brey R Brunetta P. International consensus for a definition of disease flare in lupus. Lupus. (2011) 20(5):453–62. 10.1177/0961203310388445 21148601

[14] Zen M Bassi N Nalotto L Canova M Bettio S Gatto M. Disease activity patterns in a monocentric cohort of SLE patients: a seven-year follow-up study. Clin Exp Rheumatol. (2012) 30:856–63. 22765883

[15] Polachek A Gladman DD Su J Urowitz MB. Defining low disease activity in systemic lupus erythematosus. Arthritis Care Res (Hoboken). (2017) 69(7):997–11003. 10.1002/acr.23109 27696791

[16] Franklyn K Lau CS Navarra SV Louthrenoo W Lateef A Hamijoyo L. Definition and initial validation of a lupus low disease activity state (LLDAS). Ann Rheum Dis. (2016) 75:1615–21. 10.1136/annrheumdis-2015-207726 26458737

[17] Vollenhoven RV Voskuyl A Bertsias G Aranow C Aringer M Arnaud L. A framework for remission in SLE: consensus findings from a large international task force on definitions of remission in SLE (DORIS). Ann Rheum Dis. (2017) 76:554–61. 10.1136/annrheumdis-2016-209519 27884822

[18] Lerkvaleekul B Apiwattanakul N Tangnararatchakit K Jirapattananon N Srisala S Vilaiyuk S. Associations of lymphocyte subpopulations with clinical phenotypes and long-term outcomes in juvenile-onset systemic lupus erythematosus. PLoS One. (2022) 17(2):e0263536. 10.1371/journal.pone.0263536 35130317 PMC8820627

[19] Mokbel A Fouad NA Alkemary A Abdo M. Disease activity at the onset of diagnosis as a predictor of disease outcomes in a cohort of patients with systemic lupus erythematosus: a *post hoc* retrospective analysis of the COMOSLE-EGYPT study. Clin Rheumatol. (2025) 44(1):229–35. 10.1007/s10067-024-07222-w 39548046 PMC11729062

[20] Urowitz MB Gladman DD Ibañez D Su J Mursleen S Sayani A. Effect of disease activity on organ damage progression in systemic lupus erythematosus: university of Toronto lupus clinic cohort. J Rheumatol. (2021) 48(1):67–73. 10.3899/jrheum.190259 32238510

[21] Brunner HI Silverman ED To T Bombardier C Feldman BM. Risk factors for damage in childhood-onset systemic lupus erythematosus: cumulative disease activity and medication use predict disease damage. Arthritis Rheum. (2002) 46(2):436–44. 10.1002/art.10072 11840446

[22] Li S Xue Y Kuang W Deng J Zhang J Tan X. Age-related differences in clinical and laboratory characteristics of childhood-onset systemic lupus erythematosus: pre-puberal-onset SLE is prone to delayed diagnosis. Lupus. (2023) 32(14):1675–80. 10.1177/09612033231212522 37905512

[23] Kavrul Kayaalp G Esencan D Guliyeva V Arık SD Türkmen Ş Şahin S. Childhood-onset systemic lupus erythematosus: a descriptive and comparative study of clinical, laboratory, and treatment characteristics in two populations. Lupus. (2024) 33(10):1130–8. 10.1177/09612033241265975 39037381 PMC11405132

[24] Cann MP Sage AM McKinnon E Lee SJ Tunbridge D Larkins NG. Childhood systemic lupus erythematosus: presentation, management and long-term outcomes in an Australian cohort. Lupus. (2022) 31(2):246–55. 10.1177/09612033211069765 35037500

[25] Lai CC Sun YS Chen WS Liao HT Chen MH Tsai CY. Risk factors for mortality in systemic lupus erythematosus patients: analysis of adult and pediatric cohorts in Taiwan. J Chin Med Assoc. (2022) 85(11):1044–50. 10.1097/JCMA.0000000000000783 36343272 PMC12755328

[26] Groot N Shaikhani D Teng YKO de Leeuw K Bijl M Dolhain RJEM. Long-Term clinical outcomes in a cohort of adults with childhood-onset systemic lupus erythematosus. Arthritis Rheumatol. (2019) 71(2):290–301. 10.1002/art.40697 30152151 PMC6590133

[27] Calatroni M Andrulli S Doti F Bello F De Vivo G Mastrangelo A. Long-term prognosis of lupus nephritis: comparison between pediatric, adult, and advanced age onset. Front Immunol. (2025) 16:1531675. 10.3389/fimmu.2025.1531675 40181991 PMC11966454

[28] Sato V Marques I Goldenstein P Carmo L Jorge L Titan S. Lupus nephritis is more severe in children and adolescents than in older adults. Lupus. (2012) 21(9):978–83. 10.1177/0961203312443421 22451604

[29] Chan EY Yap DY Wong WT Wong WH Wong SW Lin KY. Long-term outcomes of children and adolescents with biopsy-proven childhood-onset lupus nephritis. Kidney Int Rep. (2023) 8(1):141–50. 10.1016/j.ekir.2022.10.014 36644360 PMC9831948

[30] Kersey J Broadway D. Corticosteroid-induced glaucoma: a review of the literature. Eye. (2006) 20(4):407–16. 10.1038/sj.eye.6701895 15877093

[31] Jones R III Rhee DJ. Corticosteroid-induced ocular hypertension and glaucoma: a brief review and update of the literature. Curr Opin Ophthalmol. (2006) 17(2):163–7. 10.1097/01.icu.0000193079.55240.18 16552251

[32] Takano F Ueda K Yamada-Nakanishi Y Nakamura M. Risk factors of pediatric steroid-induced ocular hypertension. Graefes Arch Clin Exp Ophthalmol. (2025) 263(3):867–72. 10.1007/s00417-024-06669-6 39455445 PMC11953184

[33] Anderson D. The development of the trabecular meshwork and its abnormality in primary infantile glaucoma. Trans Am Ophthalmol Soc. (1981) 79:458. 7342408 PMC1312195

[34] Seth A Aggarwal A. Monitoring adverse reactions to steroid therapy in children. Indian Pediatr. (2004) 41(4):349–58. 15123863

[35] Ravelli A Duarte-Salazar C Buratti S Reiff A Bernstein B Maldonado-Velazquez MR. Assessment of damage in juvenile-onset systemic lupus erythematosus: a multicenter cohort study. Arthritis Rheum. (2003) 49(4):501–7. 10.1002/art.11205 12910556

[36] Salah S Lotfy HM Mokbel AN Kaddah AM Fahmy N. Damage index in childhood-onset systemic lupus erythematosus in Egypt. Pediatr Rheumatol Online J. (2011) 9(1):36. 10.1186/1546-0096-9-36 22152340 PMC3261107

[37] Aydın D Tunce E Kavrul Kayaalp G Tüzen HI Alkan D Oğuz G. Longitudinal assessment of disease burden in juvenile systemic lupus erythematosus: a multicenter study of activity and damage scores. Lupus. (2025) 34(13):1398–405. 10.1177/09612033251386091 41021284

[38] Nikolopoulos D Loukogiannaki C Sentis G Garantziotis P Manolakou T Kapsala N. Disentangling the riddle of systemic lupus erythematosus with antiphospholipid syndrome: blood transcriptome analysis reveals a less-pronounced IFN-signature and distinct molecular profiles in venous versus arterial events. Ann Rheum Dis. (2024) 83(9):1132–43. 10.1136/ard-2024-225664 38609158 PMC11420729

[39] Nikolopoulos D Cetrez N Lindblom J Parodis I. Neuropsychiatric involvement in systemic lupus erythematosus contributes to organ damage beyond the nervous system: a post-hoc analysis of 5 phase III randomized clinical trials. Rheumatol Int. (2024) 44:1679–89. 10.1007/s00296-024-05667-5 39115551 PMC11343782

[40] Bandeira M Buratti S Bartoli M Gasparini C Breda L Pistorio A. Relationship between damage accrual, disease flares and cumulative drug therapies in juvenile-onset systemic lupus erythematosus. Lupus. (2006) 15(8):515–20. 10.1191/0961203306lu2316oa 16942004

[41] Doria A Shoenfeld Y Wu R Gambari P Puato M Ghirardello A. Risk factors for subclinical atherosclerosis in a prospective cohort of patients with systemic lupus erythematosus. Ann Rheum Dis. (2003) 62(11):1071–7. 10.1136/ard.62.11.1071 14583570 PMC1754370

[42] Gladman DD Urowitz MB Rahman P Ibañez D Tam L-S. Accrual of organ damage over time in patients with systemic lupus erythematosus. J Rheumatol. (2003) 30(9):1955–9. 12966597

[43] Zonana-Nacach A Barr SG Magder LS Petri M. Damage in systemic lupus erythematosus and its association with corticosteroids. Arthritis Rheum. (2000) 43(8):1801–8. 10.1002/1529-0131(200008)43:8<1801::AID-ANR16>3.0.CO;2-O 10943870 10.1002/1529-0131(200008)43:8<1801::AID-ANR16>3.0.CO;2-O

[44] Khan A Shamim R Wagan AA Khan SM Ahmed SN Haroon M. Avascular necrosis in systemic lupus erythematosus patients: analysis of the demographics, clinical manifestations, management and outcomes. Egypt Rheumatol. (2023) 45(3):261–5. 10.1016/j.ejr.2023.05.002

[45] Kunyakham W Foocharoen C Mahakkanukrauh A Suwannaroj S Nanagara R. Prevalence and risk factor for symptomatic avascular necrosis development in Thai systemic lupus erythematosus patients. Asian Pac J Allergy Immunol. (2012) 30(2):152. 22830295

[46] Sayarlioglu M Yuzbasioglu N Inanc M Kamali S Cefle A Karaman O. Risk factors for avascular bone necrosis in patients with systemic lupus erythematosus. Rheumatol Int. (2012) 32(1):177–82. 10.1007/s00296-010-1597-9 20711782

[47] Chinnadurai S Chilukuri B Mahendran B Mantharam V Selvakumar B Sankaralingam R. Clinical profile of osteonecrosis in systemic lupus erythematosus–experience from a tertiary care centre in south India. J Family Med Prim Care. (2020) 9(8):4363–7. 10.4103/jfmpc.jfmpc_1234_19 33110861 PMC7586507

[48] Shaharir SS Chua SH Mohd R Mustafar R Noh MM Shahril NS. Risk factors for symptomatic avascular necrosis (AVN) in a multi-ethnic systemic lupus erythematosus (SLE) cohort. PLoS One. (2021) 16(3):e0248845. 10.1371/journal.pone.0248845 33739994 PMC7978335

[49] Tse SM Mok CC. Time trend and risk factors of avascular bone necrosis in patients with systemic lupus erythematosus. Lupus. (2017) 26(7):715–22. 10.1177/0961203316676384 27831540

[50] Al Saleh J El Sayed M Salah N Harb D Khan N Mohammed N. Predictors of avascular necrosis of the hip in Emiratis patients with systemic lupus erythematosus. Egypt J Immunol. (2010) 17(1):29–40. 22053607

[51] Assouline-Dayan Y Chang C Greenspan A Shoenfeld Y Gershwin ME. Pathogenesis and natural history of osteonecrosis. Semin Arthritis Rheum. (2002) 32(2):94–124. 10.1053/sarh.2002.33724b 12430099

[52] Faezi ST Hoseinian AS Paragomi P Akbarian M Esfahanian F Gharibdoost F. Non-corticosteroid risk factors of symptomatic avascular necrosis of bone in systemic lupus erythematosus: a retrospective case-control study. Mod Rheumatol. (2015) 25(4):590–4. 10.3109/14397595.2014.987366 25528860

[53] Cheng C Huang C Chen Z Zhan F Duan X Wang Y. Risk factors for avascular necrosis in patients with systemic lupus erythematosus: a multi-center cohort study of Chinese SLE treatment and research group (CSTAR) registry XXII. Arthritis Res Ther. (2023) 25(1):78. 10.1186/s13075-023-03061-3 37173771 PMC10176939

[54] Chang C Greenspan A Gershwin ME. The pathogenesis, diagnosis and clinical manifestations of steroid-induced osteonecrosis. J Autoimmun. (2020) 110:102460. 10.1016/j.jaut.2020.102460 32307211

[55] Taietti I Zini F Conti EA Cristini E Borzani I Ramponi G. Avascular necrosis in pediatric rheumatic diseases: an Italian retrospective multicentre study. Ital J Pediatr. (2025) 51(1):20. 10.1186/s13052-025-01845-8 39876000 PMC11776117

[56] Zhu K-K Xu W-D Pan H-F Zhang M Ni J Ge F-Y. The risk factors of avascular necrosis in patients with systemic lupus erythematosus: a meta-analysis. Inflammation. (2014) 37(5):1852–64. 10.1007/s10753-014-9917-y 24862229

[57] Birla V Vaish A Vaishya R. Risk factors and pathogenesis of steroid-induced osteonecrosis of femoral head-A scoping review. J Clin Orthop Trauma. (2021) 23:101643. 10.1016/j.jcot.2021.101643 34722150 PMC8531658

[58] Sit AKK Chan WKY. Risk factors for damage in childhood-onset systemic lupus erythematosus in asians: a case control study. Pediatr Rheumatol Online J. (2018) 16:56. 10.1186/s12969-018-0271-8 30201026 PMC6131800

[59] Petri M Magder LS. Comparison of remission and lupus low disease activity state in damage prevention in a United States systemic lupus erythematosus cohort. Arthritis Rheumatol. (2018) 70(11):1790–5. 10.1002/art.40571 29806142 PMC6203602

[60] Zen M Iaccarino L Gatto M Saccon F Larosa M Ghirardello A. Lupus low disease activity state is associated with a decrease in damage progression in Caucasian patients with SLE, but overlaps with remission. Ann Rheum Dis. (2018) 77(1):104–10. 10.1136/annrheumdis-2017-211613 28970217

[61] Tani C Vagelli R Stagnaro C Carli L Mosca M. Remission and low disease activity in systemic lupus erythematosus: an achievable goal even with fewer steroids? Real-life data from a monocentric cohort. Lupus Sci Med. (2018) 5(1):e000234. 10.1136/lupus-2017-000234 29531772 PMC5844382

[62] Tsang-A-Sjoe MW Bultink IE Heslinga M Voskuyl AE. Both prolonged remission and lupus low disease activity state are associated with reduced damage accrual in systemic lupus erythematosus. Rheumatology. (2017) 56:121–8. 10.1093/rheumatology/kew377 27803306

[63] Piga M Floris A Cappellazzo G Chessa E Congia M Mathieu A. Failure to achieve lupus low disease activity state (LLDAS) six months after diagnosis is associated with early damage accrual in Caucasian patients with systemic lupus erythematosus. Arthritis Res Ther. (2017) 19(1):247. 10.1186/s13075-017-1451-5 29126432 PMC5681839

[64] Na Nakorn K Piyaphanee N Sukharomana M Pinpatanapong R Charuvanij S. Outcomes of achieving lupus low disease activity state and damage accrual in childhood-onset systemic lupus erythematosus. Clin Rheumatol. (2023) 42:1655–64. 10.1007/s10067-023-06533-8 36780064

