# Supplementary material for: Impact of high disease activity on damage accrual and disease outcomes in childhood-onset systemic lupus erythematosus
Source: Front Pediatr. 2026 Jun 18;14:1765082. doi: 10.3389/fped.2026.1765082 (PMC13323140; doi:10.3389/fped.2026.1765082)
Supplement: Supplementary file 1 [file Datasheet1.docx]

Supplementary Material

Impact of high disease activity on damage accrual and

disease outcomes in childhood-onset systemic lupus erythematosus

Ninlapat Jidmahawong, Butsabong Lerkvaleekul, Kwanchai Pirojsakul, Soamarat Vilaiyuk^*^

*** Correspondence:** Soamarat Vilaiyuk: [soamarat21@hotmail.com](mailto:soamarat21@hotmail.com)

**1. Calculation method of time-adjusted mean SLEDAI-2K**

- 1. Formula

Time-adjusted mean SLEDAI-2K = Total cumulative SLEDAI-2K over time

Total follow-up time

- 1. Calculation of cumulative SLEDAI-2K by using the trapezoidal rule

Cumulative SLEDAI-2K = Sum of SLEDAI-2K_i_ + SLEDAI-2K_i+1_

x (*t*_i+1_–*t*_i_)

2

*t_n_*–*t*_1_

SLEDAI-2K_i_ = SLEDAI-2K score at visit i

*t*_i_ = time at visit i

(*t*_i+1_–*t*_i_) = time interval between visits

*t_n_*–*t*_1_ = total follow-up duration

**2. Calculation method of time-adjusted cumulative high disease activity status (cHDAS)**

- 1. General formula

For two consecutive visits:

- $t_{1}$: time of the first visit
- $t_{2}$: time of the second visit
- $S_{1}$: SLEDAI-2K at $t_{1}$
- $S_{2}$: SLEDAI-2K at $t_{2}$
- $S_{\text{threshold}}$: HDAS threshold (≥10)

The time at which SLEDAI-2K crosses the HDAS threshold ($t^{*}$) was estimated using linear interpolation:

*t** = *t*_1_ + (*t*_2_ – *t*_1_) x S_threshold_ (10) – S_1_

S_2_ – S_1_

2.2 Application for HDAS duration

The duration of HDAS within each interval was calculated as follows:

1. No change in HDAS status

- If S_1_ ≥ 10 and S_2_ ≥ 10 HDAS duration = *t*_2_ – *t*_1_
- If S1 < 10 and S2 < 10 HDAS duration = 0

### Crossing from HDAS to non-HDAS

- If S1 ≥ 10 and S2 < 10 HDAS duration = *t**– *t*_1_

### Crossing from non-HDAS to HDAS

- If S1 < 10 and S2 ≥ 10 HDAS duration = *t*_2_ – *t**

## 2.3 Total cumulative HDAS

The cumulative HDAS (cHDAS) was calculated as: Sum of HDAS duration across all intervals

2.4 Time-adjusted cHDAS

To account for varying follow-up duration:

Time-adjusted cHDAS (%) = cHDAS

X 100

Total follow-up time

**1. Supplementary Figure and Tables**

**1.1 Supplementary Tables**

**Supplementary Table 1.** Details of disease damage by organ/system.

| **Disease damage by organ/system** | **Frequency (%)** | **HDAS (%)** | **Non-HDAS (%)** |
| --- | --- | --- | --- |
| **Ocular** | 16 | 14 | 2 |
| - Cataract - Glaucoma | 13 (81.25)  2 (12.5) | 12 (85.7)  1 (7.1) | 1 (50)  1 (50) |
| - Retinal scar - Central scotoma | 1 (6.25)  1 (6.25) | 1 (7.1)  1 (7.1) | 0  0 |
| **Neuropsychiatric** | 16 | 13 | 3 |
| - Cognitive impairment or major psychosis | 6 (37.5) | 5 (38.5) | 1 (33.3) |
| - Seizures requiring therapy for 6 months | 7 (43.8) | 6 (46.2) | 1 (33.3) |
| - Cerebrovascular accident ever | 6 (37.5) | 5 (38.5) | 1 (33.3) |
| - Cranial or peripheral neuropathy | 0 | 0 | 0 |
| - Transverse myelitis | 0 | 0 | 0 |
| **Musculoskeletal** | 14 | 12 | 2 |
| - Muscle atrophy or weakness - Deforming or erosive arthritis - Osteoporosis with fracture or vertebral collapse - Avascular necrosis - Osteomyelitis | 0  1 (7.1)  6 (42.9)  8 (57.1)  0 | 0  1 (8.3)  6 (50)  6 (50)  0 | 0  0  0  2 (100)  0 |
| **Renal** | 2 | 2 | 0 |
| - Estimated or measured GFR < 50% | 0 | 0 | 0 |
| - Proteinuria | 0 | 0 | 0 |
| - End stage renal disease | 2 (100) | 2 (100) | 0 |
| **Pulmonary** | 3 | 3 | 0 |
| - Pulmonary hypertension | 1 (33.3) | 1 (33.3) | 0 |
| - Pulmonary fibrosis | 0 | 0 | 0 |
| - Shrinking lung | 2 (66.7) | 2 (66.7) | 0 |
| - Pleural fibrosis | 0 | 0 | 0 |
| - Pulmonary infarction | 0 | 0 | 0 |
| **Cardiovascular** | 0 | 0 | 0 |
| - Angina or coronary artery bypass | 0 | 0 | 0 |
| - Myocardial infarction ever | 0 | 0 | 0 |
| - Cardiomyopathy (ventricular dysfunction) | 0 | 0 | 0 |
| - Valvular disease | 0 | 0 | 0 |
| - Pericarditis for 6 months | 0 | 0 | 0 |
| **Peripheral vascular** | 1 | 1 | 0 |
| - Claudication for 6 months | 0 | 0 | 0 |
| - Minor tissue loss | 0 | 0 | 0 |
| - Significant tissue loss ever | 0 | 0 | 0 |
| - Venous thrombosis with swelling, ulceration or venous stasis | 1 (100) | 1 (100) | 0 |
| **Gastrointestinal** | 0 | 0 | 0 |
| - Infarction or resection of bowel, spleen, liver or gall bladder ever | 0 | 0 | 0 |
| - Mesenteric insufficiency | 0 | 0 | 0 |
| - Chronic peritonitis | 0 | 0 | 0 |
| - Stricture or upper gastrointestinal tract surgery ever | 0 | 0 | 0 |
| - Pancreatic insufficiency requiring enzyme replacement or with pseudocyst | 0 | 0 | 0 |
| **Musculoskeletal** | 14 | 12 | 2 |
| - Muscle atrophy or weakness | 0 | 0 | 0 |
| - Deforming or erosive arthritis | 1 (7.1) | 1 (8.3) | 0 |
| - Osteoporosis with fracture or vertebral collapse | 5 (35.7) | 5 (41.7) | 0 |
| - Avascular necrosis | 8 (57.1) | 6 (50) | 2 (100) |
| - Osteomyelitis | 0 | 0 | 0 |
| **Skin** | 2 | 0 | 2 |
| - Scarring chronic alopecia | 2 (100) | 0 | 2 (100) |
| - Extensive scarring or panniculum other than scalp | 0 | 0 | 0 |
| - Skin ulceration for > 6 months | 0 | 0 | 0 |
| **Premature gonadal failure** | 1 (100) | 1 (100) | 0 |
| **Diabetes** | 5 (100) | 5 (100) | 0 |
| **Malignancy** | 1 (100) | 1 (100) | 0 |

**Supplementary Table 2.** Kaplan–Meier analysis of time to organ damage according to time-adjusted cumulative prednisolone dose.

| Time-adjusted cumulative prednisolone dose (mg/kg/day) | Mean time to organ damage (years) | Median time to organ damage (years) | *p*-value |
| --- | --- | --- | --- |
| > 0.15 | 9.4 (95% CI 7.9-10.9) | - | < 0.001* |
| > 0.2 | 8.8 (95% CI 7.2-10.4) | 7.3 (95% CI 6.1-8.6) | < 0.001* |
| > 0.25 | 6.0 (95% CI 5.0-7.0) | 6.6 (95% CI 4.2-9.0) | < 0.001* |
| > 0.3 | 4.8 (95% CI 3.8-5.8) | 5.1 (95% CI 1.3-8.9) | < 0.001* |
| > 0.4 | 4.2 (95% CI 3.0-5.4) | 2.3 (95% CI 1.3-3.4) | < 0.001* |
| > 0.5 | 3.9 (95% CI 2.4-5.3) | 1.5 (95% CI 0.8-2.2) | < 0.001* |

*A *p* < 0.05 was considered statistically significant.

**Supplementary Table 3.** The association between time-adjusted cumulative prednisolone dose and damage accrual (SDI ≥1).

| Time-adjusted cumulative prednisolone dose (mg/kg/day) | Hazard Ratio (95%CI) | *p*-value |
| --- | --- | --- |
| > 0.15 | 3.9 (1.7-9.3) | <0.002* |
| > 0.2 | 4.1 (2.0-8.4) | <0.001* |
| > 0.25 | 4.7 (2.5-8.9) | <0.001* |
| > 0.3 | 6.7 (3.6-12.4) | <0.001* |
| > 0.4 | 8.9 (4.8-16.2) | <0.001* |
| > 0.5 | 8.0 (4.4-14.4) | <0.001* |

*A *p* < 0.05 was considered statistically significant. SDI, Systemic Lupus International Collaborating Clinics/American College of Rheumatology Damage Index.
